# A Substrate-Integrated Waveguide Filtering Power Divider with Broadside-Coupled Inner-Meander-Slot Complementary Split-Ring Resonator

**DOI:** 10.3390/mi17010103

**Published:** 2026-01-13

**Authors:** Jinjia Hu, Chen Wang, Yongmao Huang, Shuai Ding, Maurizio Bozzi

**Affiliations:** 1 School of Electrical and Electronic Information, Xihua University, Chengdu 610039, China; 2 School of Physics, University of Electronic Science and Technology of China, Chengdu 611731, China; uestcding@uestc.edu.cn; 3 Department of Computers, Electrical and Biomedical Engineering, University of Pavia, 27100 Pavia, Italy; maurizio.bozzi@unipv.it

**Keywords:** broadside-coupling, complementary split-ring resonator (CSRR), filtering power divider, meander-shaped slot, substrate integrated waveguide (SIW)

## Abstract

In this work, a substrate-integrated waveguide (SIW) filtering power divider with a modified complementary split-ring resonator (CSRR) is reported. Firstly, by integrating the meander-shaped slots with the conventional CSRR, the proposed inner-meander-slot CSRR (IMSCSRR) can enlarge the total length of the defected slot and increase the width of the split, thus enhancing the equivalent capacitance and inductance. In this way, the fundamental resonant frequency of the IMSCSRR can be effectively decreased without enlarging the circuit size, which can generally help to reduce the physical size by over 35%. Subsequently, to further reduce the circuit size, two IMSCSRRs are separately loaded on the top and bottom metal covers to constitute a broadside-coupled IMSCSRR, which is combined with the SIW. To verify the efficacy of the proposed SIW-IMSCSRR unit cell, a two-way filtering power divider is implemented. It combines the band-selection function of a filter and the power-distribution property of a power divider, thereby enhancing system integration and realizing size compactness. Experimental results show that the proposed filtering power divider achieves a center frequency of 3.53 GHz, a bandwidth of about 320 MHz, an in-band insertion loss of (3 + 1.3) dB, an in-band isolation of over 21 dB, and a size reduction of about 30% compared with the design without broadside-coupling, as well as good magnitude and phase variations. All the results indicate that the proposed filtering power divider achieves a good balance between low loss, high isolation, and compact size, which is suitable for system integration applications in microwave scenarios.

## 1. Introduction

Substrate-integrated waveguide (SIW) is a popular guided-wave structure developed over the past two and a half decades. It integrates several features of the conventional rectangular waveguide, like high quality factor, low loss, and high power handling capacity. Meanwhile, it also possesses planarization and system integration characteristics of other planar guided-wave structures, including microstrip, coplanar waveguide, and slot line [1]. Therefore, SIW has been utilized to design microwave filters [2], diplexers [3], power dividers [4], antennas [5], and many others. However, owing to the inherent cutoff frequency limitation of its dominant transmissible mode, SIW generally has larger physical size compared to its corresponding microstrip and coplanar waveguide counterparts, which restricts its practical application in microwave systems. To overcome this drawback, various miniaturization techniques for SIW have been developed. First of all, the fractional-mode SIW enabled by virtual equivalent magnetic wall cutting is an effective method to reduce size. With a single virtual magnetic wall cutting, SIW is divided into half-mode SIW (HMSIW) with a similar cutoff frequency but only half of the size. With multiple virtual magnetic wall cuttings in an SIW cavity, various fractional-mode SIW cavities, such as quarter-mode SIW (QMSIW) and eighth-mode SIW (EMSIW), separately with 75% and 87.5% size reductions, can be achieved. The second size miniaturization approach is utilizing multilayer structures. For instance, folding SIW into two or more layers along the longitudinal direction is useful to reduce the transverse size. Meanwhile, owing to the contribution from the ridge to its equivalent capacitance, the ridged SIW can effectively miniaturize size as well. Moreover, cross-layer coupling topology can squeeze the planar size of the SIW and improve its system integration. Subsequently, another method employs defected electromagnetic structures, such as defected ground structure, electromagnetic bandgap structures, or complementary split-ring resonators (CSRRs), in SIW. Loading defected electromagnetic structures on the surface metal of SIW will rearrange the surface current distributions and eventually influence the equivalent inductance and capacitance. In this way, size reduction can be expected. For instance, an HMSIW ultra-wideband filter with reconfigurable notched bands is proposed with compact size, which is mainly contributed by the utilization of three U-shaped slots on the top metal [6]. In [7], CSRRs are loaded into the SIW to realize evanescent mode transmission and design compact planar filters. Particularly, such configurations can generate a passband below the dominant cutoff frequency of SIW, which provides notable size miniaturization. Furthermore, employing the slow-wave effect is also an effective method to reduce the size of SIW. In [8], mender-shaped microstrip polylines are patterned on SIW to lengthen the signal path and ultimately generate the slow-wave effect. Using this slow-wave SIW (SWSIW), a multichannel filtering crossover is implemented with a size reduction of over 50%. In fact, with the patterned mender-shaped microstrip polylines, the equivalent inductance can be enhanced effectively, while the equivalent capacitance is influenced only slightly. Hence, the corresponding equivalent permeability can be enlarged remarkably without influencing the equivalent permittivity. Therefore, the phase constant of SWSIW’s dominant mode is enlarged and its physical size is decreased as well.

On the other hand, to simultaneously enhance system integration and save circuit size, multifunction SIW components have been proposed. For instance, in [5], the frequency-selection property is integrated with the radiation function in a single filtering antenna. Meanwhile, both filter and crossover are realized in a single SWSIW cavity [8]. By combining the functions of a filter and a power divider, filtering power divider exhibit good potential for system integration and size miniaturization. A dual-mode SIW filtering power divider is proposed to design a parallel feedback oscillator with low phase noise in [9]. Different vias are drilled at various locations in a circular SIW cavity to build proper perturbations and excite the TM010 and TM110 modes. Then, with symmetrical two-output feedings, the filtering and power-dividing functions can be realized simultaneously. Unfortunately, using higher-order modes generally results in bulky circuit size. In [10], a balanced filtering power divider is developed based on a triangular SIW cavity. It utilizes the TE120 mode to transmit the differential mode and realize a common-mode suppression of 42 dB, as well as a transmission zero in the stopband. However, even with the isolated resistor, the isolation between its output ports only reaches 16 dB. Generally, compared to a single-mode cavity, a multi-mode cavity can achieve compact size and wider operation bandwidth, which exhibits quite good potential for filtering power divider design. In 2024, a broadband filtering power divider with a quintuple-mode SIW cavity was designed [11]. By tuning the locations and size of the four perturbed slots, the distribution of the five eigenmodes can be adjusted flexibly, and a fractional bandwidth (FBW) of 48.6% is achieved. However, the filtering and isolation performance are not specifically considered in this design. In [12], a balanced-to-single filtering power divider with out-of-phase operation is developed based on a square SIW cavity for the application of a filtering antenna array. It utilizes the orthogonality between the TE102 and TE201 modes, combined with differential feedings. Moreover, it is designed with a center frequency of 5.2 GHz and a bandwidth of 180 MHz. Subsequently, it is worth to note that introducing defected electromagnetic structures into SIW can contribute to generating filtering functions and reducing physical size. For instance, an unequal SIW filtering power divider loaded with CSRRs is utilized to realize a decoupling network for an array antenna in [13]. Owing to the interaction between SIW and CSRRs, the filtering power divider achieves quite a compact size. Meanwhile, by adjusting the positions of the output microstrip lines, a 1:4 division ratio is obtained. However, its isolation is not optimal. Simultaneously, a microfluidically reconfigurable SIW power divider is developed using a three-dimensional (3D) printing process [14]. With microfluidic channels for distilled water, the equivalent permittivity of the SIW section can be tuned, and the operation frequency can be shifted easily. In addition, a lumped resistor is employed to enhance the isolation to over 15 dB. Furthermore, a compact five-way filtering power divider is designed in [15]. It utilizes the multi-cavity coupling topology to save size and utilizes the short-circuited vias and U-shaped slots to adjust stopband, thereby tuning the bandwidth. Nevertheless, its isolation performance is limited as well. In [16], a frequency-reconfigurable nonreciprocal SIW filtering power divider is presented. It employs a common SIW cavity under degenerate mode operation to miniaturize circuit size and utilizes three lumped resistors to improve the isolation to 20 dB. Finally, two-way and four-way SIW filtering power divider using a multi-port division network are reported in [17]. Although the power divisions can be adjusted flexibly and the selectivity is quite good, their sizes are bulky due to the non-resonant cavity.

In this work, a compact SIW filtering power divider with an inner-meander-slot CSRR (IMSCSRR) is presented. The proposed IMSCSRR can increase the effective length of the defected slot and enlarge the effective width of the split, thereby enhancing the equivalent capacitance and inductance, decreasing the fundamental resonant frequency, and eventually contributing to size miniaturization. Moreover, a broadside-coupling scheme is employed to include extra mutual capacitive coupling for further size reduction. With broadside-coupling scheme used in the proposed filtering power divider, the inner coupling can be enhanced, the bandwidth can be enlarged, and the transmission loss can be decreased. Hence, the proposed broadside-coupled SIW-IMSCSRR filtering power divider can achieve a good balance between transmission performance and size compactness. The work is organized as below. Section 2 describes the working principle of the proposed IMSCSRR and its application to SIW. The design procedures of the proposed broadside-coupled SIW-IMSCSRR filtering power divider are introduced in Section 3. Section 4 shows the experimental results and discussion. Finally, a conclusion is given.

## 2. SIW Unit Cell with IMSCSRR

### 2.1. Inner-Meander-Slot CSRR

**Figure 1:**
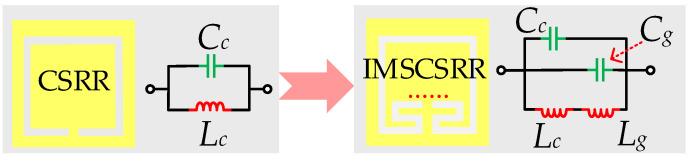
Evolution process from the conventional CSRR to the proposed IMSCSRR and their equivalent circuit models.

Figure 1 illustrates the evolution process from the conventional CSRR to the proposed IMSCSRR, as well as their simplified equivalent circuit models. Firstly, according to the theory of coupled resonators, both the conventional CSRR and the proposed IMSCSRR can be equivalent to a simple parallel resonant circuit constituted by an inductor and a capacitor. Moreover, it can be easily obtained that, for the conventional CSRR, its equivalent inductance and capacitance are denoted by *L_c_* and *C_c_*, respectively. Moreover, for the proposed IMSCSRR, *L_c_* and *C_c_* separately denote the equivalent inductance and capacitance contributed from the original CSRR section, while *L_g_* and *C_g_* separately represent the equivalent inductance and capacitance introduced by the newly added inner meander-shaped slots.

Hence, the fundamental resonant frequencies of the conventional CSRR and the proposed IMSCSRR can be separately expressed as follows:

(1) $$f_{1}=1/2\pi \sqrt{L_{c}C_{c}},$$

(2) $$f_{2}=1/2\pi \sqrt{\left(L_{c}+L_{g}\right)\left(C_{c}+C_{g}\right)},$$

Therefore, the proposed IMSCSRR alters the equivalent inductance and capacitance by etching the inner meander-shaped slots without enlarging the circuit size. Based on the aforementioned formulas, it can be concluded that, compared to the conventional CSRR, both the equivalent inductance and capacitance of the proposed IMSCSRR are effectively enhanced. Hence, the equivalent capacitance and inductance of the proposed IMSCSRR are always separately larger than the corresponding counterparts of the conventional CSRR. Therefore, the product of the equivalent inductance and capacitance of the proposed IMSCSRR is always greater than that of the conventional CSRR with the same physical size. Then, the fundamental eigenmode of the proposed IMSCSRR must operate at a lower frequency than that of the CSRR with the same size. As a result, the proposed IMSCSRR enables extra size reduction on the basis of the CSRR. Furthermore, as shown in the geometrical configuration of the IMSCSRR, with the inner meander-shaped slots, the total length of the split-ring slot is enlarged, and the equivalent capacitance is also increased. Meanwhile, with the inner meander-shaped slots, the width of the split is increased simultaneously; thus, the equivalent inductance is enhanced as well. Subsequently, as the number of bends in the inner meander-shaped slots increases, so do the equivalent capacitance *C_g_* and the equivalent inductance *L_g_*. In this way, the total capacitance (*C_c_* + *C_g_*) and the total inductance (*L_c_* + *L_g_*) are simultaneously increased. Moreover, increasing the transverse lengths of the inner meander-shaped slots is equivalent to enhancing *C_g_*, thereby enlarging the total capacitance (*C_c_* + *C_g_*).

### 2.2. SIW and HMSIW Unit Cells with Broadside-Coupled Inner-Meander-Slot CSRRs

**Figure 2:**
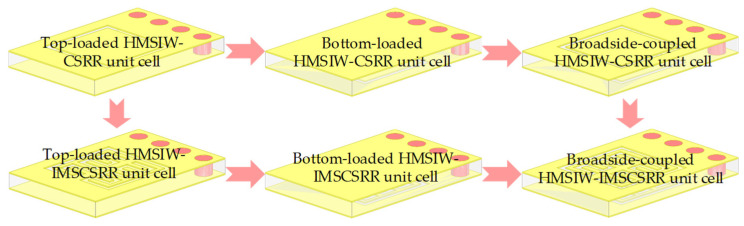
Evolution process from the HMSIW-CSRR unit cell to the broadside-coupled HMSIW-IMSCSRR unit cell.

By etching the CSRR or IMSCSRR on the top or bottom metal cover of the SIW, the SIW-CSRR and SIW-IMSCSRR unit cells are constituted. Similarly, the HMSIW-CSRR and HMSIW-IMSCSRR unit cells can also be formed. Figure 2 shows the evolution process from the conventional HMSIW-CSRR unit cell to the proposed broadside-coupled HMSIW-IMSCSRR unit cell. From Figure 2, it can be seen that the top-loaded, bottom-loaded, and broadside-coupled HMSIW-CSRR and HMSIW-IMSCSRR unit cells are presented. Due to the structural symmetry of the HMSIW along the thickness direction, loading the CSRR or IMSCSRR on the top or bottom metal cover generally produces consistent electromagnetic effects. However, loading two CSRRs or IMSCSRRs of the same size on both the top and bottom metal covers of the HMSIW, namely the broadside-coupled HMSIW-CSRR or the broadside-coupled HMSIW-IMSCSRR, will produce much different responses. In the broadside-coupled HMSIW-CSRR and HMSIW-IMSCSRR unit cells, mutual coupling is excited, and a notable mutual capacitance effect is generated between the two CSRRs or IMSCSRRs, which enhances the equivalent capacitance and consequently increases the product of the equivalent inductance and capacitance [18]. Therefore, compared to the top- and bottom-loaded HMSIW-CSRR unit cells of the same physical size, the broadside-coupled HMSIW-CSRR operates at a lower fundamental frequency. Similarly, compared to the top- and bottom-loaded HMSIW-IMSCSRR unit cells of the same physical size, the proposed broadside-coupled HMSIW-IMSCSRR unit cell operates at a further lower frequency. In this way, size miniaturization is achieved by the broadside-coupled unit cells.

To better understand the resonant characteristics of the broadside-coupled unit cells, eigenmode analysis by using a commercial full-wave electromagnetic simulator is conducted on the top-loaded SIW-CSRR and HMSIW-CSRR, the top-loaded SIW-IMSCSRR and HMSIW-IMSCSRR, the broadside-coupled SIW-CSRR and HMSIW-CSRR, and the broadside-coupled SIW-IMSCSRR and HMSIW-HMSIW unit cells. For reasonableness and convincingness of the eigenmode analysis, the eight unit cells mentioned above are configured with the same substrate, which has a thickness of 0.508 mm, a relative permittivity of 4.38, a relative permeability of 1, a dielectric loss tangent of 0.005, and a magnetic loss tangent of 0. Meanwhile, the metal covers are set as copper with a thickness of 0.035 mm and a bulk conductivity of 5.8 × 10^7^ Siemens/m.

**Figure 3:**
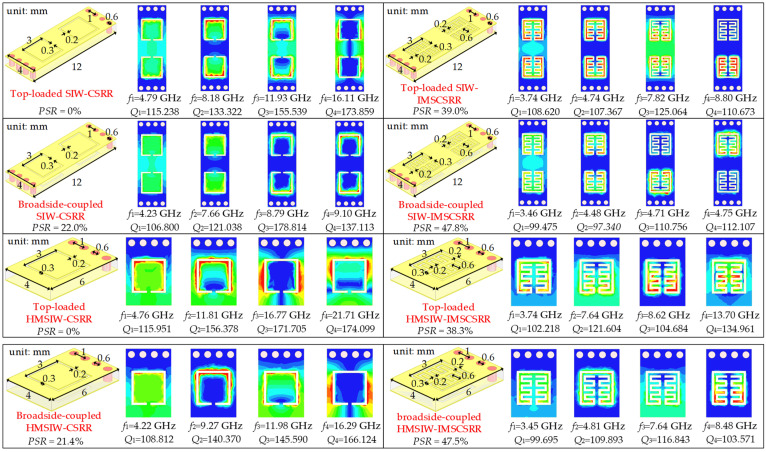
Electric filed distributions of the four lowest eigenmodes in the top-loaded SIW-CSRR and HMSIW-CSRR, top-loaded SIW-IMSCSRR and HMSIW-IMSCSRR, broadside-coupled SIW-CSRR and HMSIW-CSRR, and broadside-coupled SIW-IMSCSRR and HMSIW-IMSCSRR unit cells.

Figure 3 shows the electric field distributions of the four lowest eigenmodes in various unit cells. For the SIW ones, the fundamental eigenmodes of the top-loaded SIW-CSRR and SIW-IMSCSRR operate at 4.79 GHz and 3.74 GHz, respectively, with corresponding unloaded quality factor (*Q_u_*) values of 115.238 and 108.620. The difference between their fundamental frequencies indicates that the IMSCSRR helps to achieve a percentage of size reduction (*PSR*) of 39.0%. Moreover, the second, third, and fourth modes of the SIW-CSRR operates at frequencies ranging from 8.18 GHz to 16.11 GHz, with *Q_u_* values ranging between 133.322 and 173.859. On the contrary, the second, third, and fourth modes of the SIW-IMSCSRR operates at frequencis covering 4.74–8.80 GHz, with corresponding *Q_u_* values covering 107.367–125.064. As shown in Figure 3, the eigenmodes of the SIW-IMSCSRR exhibit smaller *Q_u_* values than the corresponding ones of the SIW-CSRR, which is mainly caused by the larger radiation loss resulting from the increased number of defected slots in the IMSCSRR. Furthermore, the four lowest eigenmodes of the broadside-coupled SIW-CSRR operate at 4.25 GHz, 7.66 GHz, 8.79 GHz, and 9.10 GHz, respectively, with corresponding *Q_u_* values ranging from 106.800 to 178.814. Meanwhile, the four lowest eigenmodes of the broadside-coupled SIW-IMSCSRR separately exhibit resonant frequencies of 3.46 GHz, 4.48 GHz, 4.71 GHz, and 4.75 GHz, respectively, with corresponding *Q_u_* values of 99.475, 97.150, 110.756, and 112.107. Therefore, compared to the top-loaded SIW-CSRR, the broadside-coupled SIW-CSRR and SIW-IMSCSRR achieve size reduction of about 22.0% and 47.8%, respectively. In particular, the broadside-coupled SIW-IMSCSRR exhibits the best size miniaturization performance, while its four lowest eigenmodes show the worst *Q_u_* owing to the largest radiation loss from the defected broadside-coupled IMSCSRR.

Subsequently, for the HMSIW cases, the fundamental eigenmodes of the top-loaded HMSIW-CSRR and broadside-coupled HMSIW-CSRR separately operate at 4.76 GHz and 4.22 GHz, while the fundamental eigenmodes of the top-loaded HMSIW-IMSCSRR and broadside-coupled HMSIW-IMSCSRR operate at 3.74 GHz and 3.45 GHz, respectively. For these four unit cells, their fundamental eigenmodes exhibit *Q_u_* values of 115.951, 108.812, 102.218, and 99.695. Compared to the top-loaded HMSIW-CSRR, the broadside-coupled HMSIW-CSRR, the top-loaded HMSIW-IMSCSRR, and the broadside-coupled HMSIW-IMSCSRR implement *PSR* values of 21.4%, 38.3%, and 47.5%, respectively. Furthermore, for the top-loaded HMSIW-CSRR unit cell, its three lowest higher-order eigenmodes operate in the frequency range of 11.81–21.71 GHz, with associated *Q_u_* values of 156.378~174.099. For the broadside-coupled HMSIW-CSRR unit cell, its three lowest higher-order eigenmodes resonate in the frequency range of 9.27 GHz to 16.29 GHz, and the corresponding *Q_u_* values range from 140.370 to 166.124. For the top-loaded HMSIW-IMSCSRR, its three lowest higher-order eigenmodes operate at 7.64 GHz, 8.62 GHz, and 13.70 GHz, respectively, with corresponding *Q_u_* values of 121.604, 104.684, and 134.961. For the broadside-coupled HMSIW-IMSCSRR unit cell, its three lowest higher-order eigenmodes separately resonate at 4.81 GHz, 7.64 GHz, and 8.48 GHz. Meanwhile, the corresponding *Q_u_* values are 109.893, 116.843, and 103.571, respectively. Hence, it can be found that, when the CSRR is modified as the IMSCSRR and loaded into the HMSIW, size reduction is realized, but the radiation loss becomes larger and *Q_u_* becomes worse. When the top-loading scheme is changed to the broadside-coupling scheme and loaded into the HMSIW, extra size miniaturization can be implemented, while the radiation loss becomes further stronger and *Q_u_* becomes further smaller.

## 3. SIW Filtering Power Divider with Broadside-Coupled IMSCSRR

**Figure 4:**
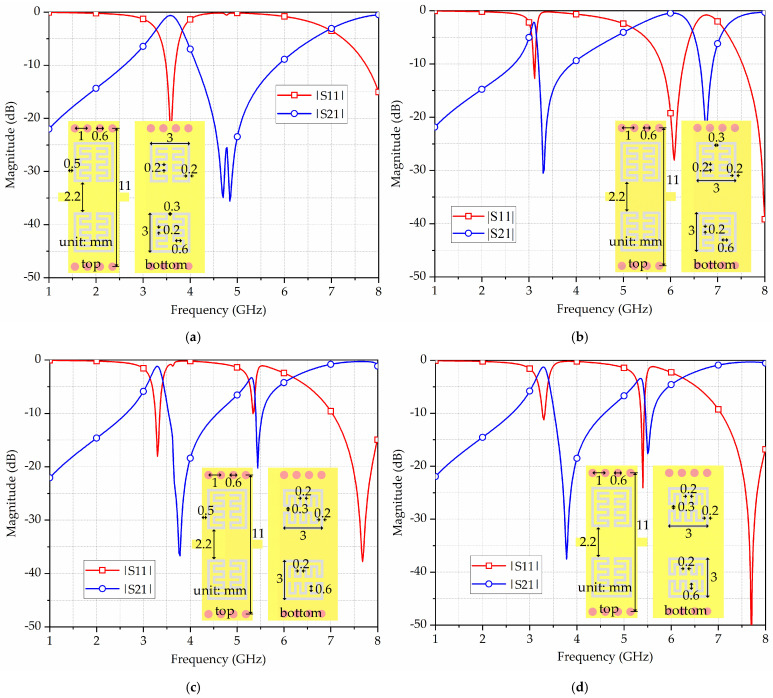
Schematic diagrams and simulation results of the broadside-coupled SIW-IMSCSRR with various orientations of the IMSCSRRs on the bottom metal cover: (**a**) face-to-face; (**b**) back-to-back; (**c**) reversely side-by-side; (**d**) equally side-by-side oriented.

To verify the availability and efficacy of the proposed broadside-coupled SIW-IMSCSRR and HMSIW-IMSCSRR, a filtering power divider is designed. Firstly, the filtering property of the broadside-coupled SIW-IMSCSRR is analyzed. Figure 4 shows the simulation results of an SIW-IMSCSRR with various orientations of the IMSCSRRs on the bottom metal cover, as well as their corresponding schematic diagrams. Similarly, for the reasonability and convincingness of the simulation, the four broadside-coupled SIW-IMSCSRR unit cells are set with the same substrate as that in Figure 3. Meanwhile, microstrip feeding lines are added at the input and output ports of the broadside-coupled SIW-IMSCSRR for the convenience of port excitation in simulation. Moreover, as illustrated in Figure 4, there are four IMSCSRRs loaded in each SIW section, two on the top metal cover and two on the bottom metal cover. Furthermore, for all four cases, the two IMSCSRRs on the top metal cover remain face-to-face oriented, while the two on the bottom metal cover are oriented variously.

According to Figure 4a, as the bottom IMSCSRRs are face-to-face oriented, the SIW-IMSCSRR exhibits a fundamental resonant frequency of 3.58 GHz and a 1 dB bandwidth of 320 MHz, with two transmission zeros at 4.7 GHz and 4.84 GHz. Meanwhile, its first parasitic passband is located above 8 GHz. From Figure 4b, as the bottom IMSCSRRs are back-to-back oriented, the fundamental eigenmode of the SIW-IMSCSRR resonates at 3.1 GHz, with a 1 dB bandwidth about 90 MHz and a transmission zero at 3.3 GHz. It exhibits two parasitic passbands located around 6.08 GHz and 8.00 GHz. In Figure 4c, as the bottom IMSCSRRs are reversely side-by-side oriented, the SIW-IMSCSRR operates at 3.3 GHz, with a 1 dB bandwidth about 160 MHz, a transmission zero at 3.78 GHz, and two parasitic passbands separately at 5.3 GHz and 7.66 GHz. Finally, as the bottom IMSCSRRs are equally side-by-side oriented, the SIW-IMSCSRR shows a fundamental resonant frequency of 3.28 GHz and a 1 dB bandwidth of 160 MHz. Moreover, its achieves a transmission zero at 3.78 GHz and two parasitic passbands at 5.36 GHz and 7.68 GHz, respectively, as shown in Figure 4d.

Furthermore, according to the four cases in Figure 4, it can be captured that, as the bottom IMSCSRRs are adjusted from face-to-face oriented to side-by-side oriented and then to back-to-back oriented, the fundamental resonant frequency of the SIW-IMSCSRR shifts lower, the passband becomes narrower, the insertion loss increases, and the location of the transmission zero becomes much lower, indicating improved selectivity. Hence, it can be predicted that the mutual coupling between the top and bottom IMSCSRRs becomes stronger with such orientation changes. However, the parasitic passbands shift much lower as well, exhibiting worse stopband performance. Therefore, the orientations of the IMSCSRRs should be set in accordance with specific demands on passband, stopband, and selectivity in practical applications. To achieve a good balance between passband and stopband performance, the bottom IMSCSRRs in the broadside-coupled SIW-IMSCSRR are supposed to be face-to-face oriented, thus obtaining a wider bandwidth and smaller insertion loss.

**Figure 5:**
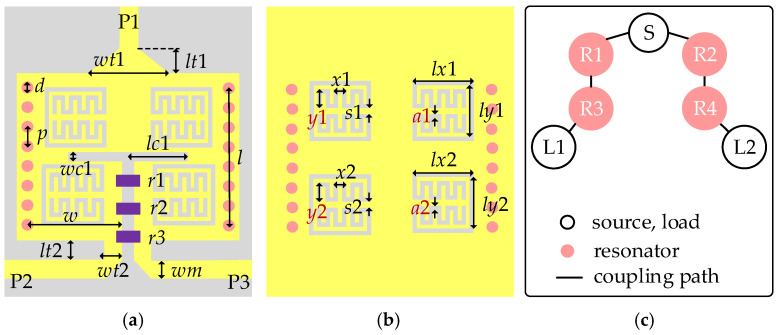
Configuration of the proposed broadside-coupled SIW-IMSCSRR filtering power divider: (**a**) top view; (**b**) bottom view; (**c**) coupling scheme.

Subsequently, based on the aforementioned analysis, a broadside-coupled SIW-IMSCSRR filtering power divider is designed, with its detailed configuration and coupling scheme given in Figure 5. As shown in Figure 5a,b, the proposed filtering power divider mainly consists of a broadside-coupled SIW-IMSCSRR, two broadside-coupled HMSIW-IMSCSRR, a tapered microstrip-to-SIW transition, two stepped microstrip-to-HMSIW transitions, and three 50 ohm microstrip lines at the input and output ports. The stepped microstrip-to-HMSIW transitions, tapered microstrip-to-SIW transition, and the input and output 50 ohm microstrip lines are included mainly for the convenience of connections to the outside coaxial cables in measurement. Meanwhile, three lumped resistors are utilized to improve the isolation. Moreover, as shown in Figure 5c, the coupling between the input transition and the broadside-coupled SIW-IMSCSRR and the coupling between the output transition and the HMSIW-IMSCSRR are both realized by the edge metal strips of the SIW area. Then, the coupling between the broadside-coupled SIW-IMSCSRR and HMSIW-IMSCSRR is realized by the middle metal strip and the slot. By tuning the width and length of the coupling slots, the coupling strength between the broadside-coupled resonators can be adjusted accordingly. Obviously, the proposed filtering power divider shows a symmetrical configuration.

Afterwards, according to the theory of coupled resonators, the internal coupling coefficient (*k*) and the external quality factor (*Q_e_*) are the two key factors in filter design [19]. For a cascaded-coupled resonator filter, the coupling elements *M_i_*_, *i*+1_, the input impedance *R*_1_, and the output impedance *R_n_* are given by (3) and (4), respectively [20,21]:

(3) $$M_{i,i+1}=1/\sqrt{g_{i}g_{i+1}},(i=1,2,\ldots ,n-1),$$

(4) $$R_{1}=1/\left(g_{0}g_{1}\right),R_{n}=1/\left(g_{n}g_{n+1}\right),$$

Here, *g_i_* (*i* = 0, 1, 2, …, *n* + 1) denote the element values of the normalized low-pass prototype filter. Based on (3) and (4), the normalized coupling matrix of the filter can be obtained. The values of *k* and *Q_e_* of the filter can be determined as follows [20,21]:

(5) $$k=\frac{1}{2}(\frac{f_{o2}}{f_{o1}}+\frac{f_{o1}}{f_{o2}})\sqrt{{\left(\frac{f_{p2}^{2}-f_{p1}^{2}}{f_{p2}^{2}+f_{p1}^{2}}\right)}^{2}-{\left(\frac{f_{o2}^{2}-f_{o2}^{2}}{f_{o2}^{2}+f_{o1}^{2}}\right)}^{2}},$$

(6) $$Q_{e1}=\frac{g_{0}g_{1}}{FBW},Q_{en}=\frac{g_{n}g_{n+1}}{FBW},$$

Here, *f_o_*_1_ and *f_o_*_2_ denote the resonant frequencies of the two individual cavities, *f_p_*_1_ and *f_p_*_2_ are the resonant frequencies of the coupled cavities, and *FBW* is the fractional bandwidth of the filter. In the proposed filtering power divider, the *FBW* is 10% with a passband ripple of 0.1 dB. Then, the normalized element values are *g*_0_ = 1.0 and *g*_1_ = 0.8430 [21]. Later, based on (5) and (6), the coupling matrix and *Q_e_* of the proposed broadside-coupled SIW-IMSCSRR filtering power divider are calculated as follows:

(7) $$k=\left[\begin{array}{cc} 0 & 0.0871\\ 0.0871 & 0 \end{array}\right],$$

(8) $$Q_{e}=10.5375$$

**Figure 6:**
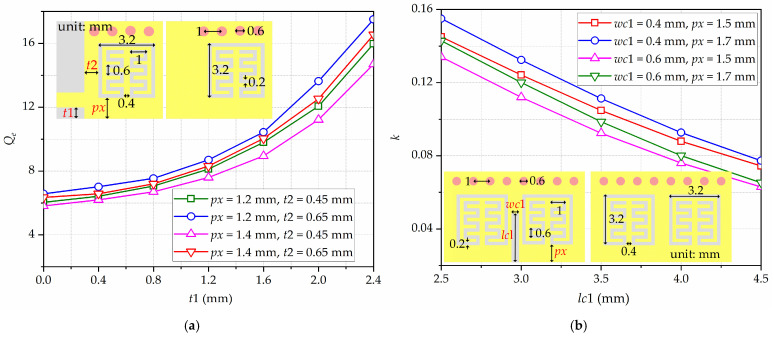
Simulated results of Qe and k of the proposed broadside-coupled HMSIW-IMSCSRR: (**a**) Qe; (**b**) k.

Thereafter, the relationships between *Q_e_* and some geometric parameters, and between *k* and other geometric parameters, are investigated with full-wave electromagnetic simulation. Here, the simulation analysis of *Q_e_* and *k* are mainly conducted on the HMSIW-IMSCSRR, based on the configuration given in Figure 5. Figure 6a shows the simulated results of *Q_e_*, which are primarily influenced by the geometric parameters *px*, *t*1, and *t*2. It can be observed that, as *t*1 becomes larger, *Q_e_* becomes larger as well. As *t*2 becomes larger, *Q_e_* also becomes larger. Whereas, as *px* becomes larger, *Q_e_* becomes smaller. Moreover, Figure 6b gives the simulated values of *k*, which are mainly related to the geometric parameters *lc*1, *wc*1, and *px*. From Figure 6b, as *px* becomes larger, *k* becomes larger as well. As *lc*1 or *wc*1 becomes larger, *k* becomes smaller. Based on the calculated values from (7) and (8) and the numerical simulations in Figure 6, the initial values of the geometrical parameters of the proposed filtering power divider can be selected.

**Figure 7:**
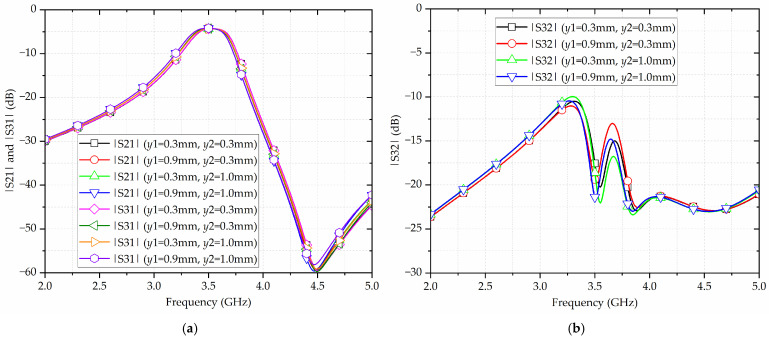
Simulated results of the proposed broadside-coupled SIW-IMSCSRR filtering power divider with various lengths of meander-shaped slots y1 and y2: (**a**) |S21| and |S31|; (**b**) |S32|.

Furthermore, some extra numerical simulations have been carried out to show the influence of the dimensions of the mender-shaped slots on the performance of the proposed filtering power divider. Figure 7 shows the simulated |S21|, |S31|, and |S32| of the proposed filtering power divider. As shown in Figure 7a, with the lengths of the meander-shaped slots *y*1 and *y*2 varying, the center frequency and bandwidth of the proposed filtering power divider change accordingly. As *y*1 increases from 0.3 mm to 0.9 mm and *y*2 increases from 0.3 mm to 1.0 mm, the center frequency shifts from 3.535 GHz to 3.495 GHz, while the bandwidth increases from 280 MHz to 310 MHz, corresponding to the FBW increasing from 7.92% to 8.87%. Hence, as *y*1 or *y*2 lengthens, the product of the equivalent capacitance and inductance of the IMSCSRR is enlarged, and thus the center frequency decreases. Meanwhile, the widening of the bandwidth indicates that the coupling coefficient increases. Moreover, according to Figure 7b, as *y*1 increases, the in-band isolation of the proposed broadside-coupled SIW-IMSCSRR filtering power divider becomes worse. On the contrary, as *y*2 increases, its in-band isolation becomes better. However, due to the contribution of the isolated resistors, the in-band isolation can always be maintained above 15 dB.

**Figure 8:**
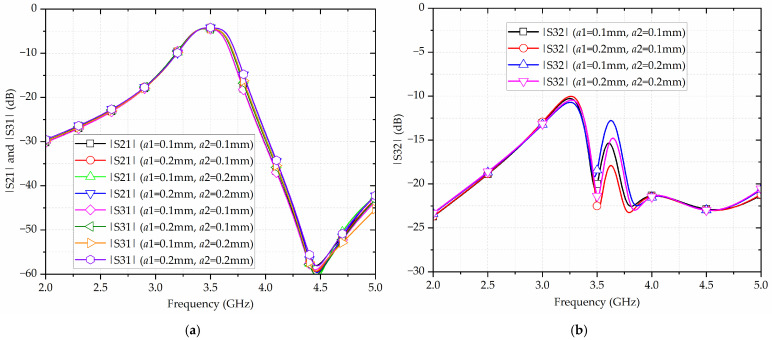
Simulated results of the proposed broadside-coupled SIW-IMSCSRR filtering power divider with various widths of meander-shaped slots a1 and a2: (**a**) |S21| and |S31|; (**b**) |S32|.

Subsequently, Figure 8 gives the simulated results of the proposed broadside-coupled SIW-IMSCSRR filtering power divider with various widths of meander-shaped slots *a*1 and *a*2. According to Figure 8a, as *a*1 and *a*2 enlarge from 0.1 mm to 0.2 mm, the center frequency shifts upwards from 3.465 GHz to 3.5 GHz, and the bandwidth increases from 250 MHz to 300 MHz, corresponding to the fractional bandwidth slightly increasing from 7.2% to 8.6%. Therefore, as *a*1 and *a*2 becomes larger, the product of the equivalent capacitance and inductance becomes smaller, and consequently the center frequency moves higher. Simultaneously, with *a*1 and *a*2 becoming larger, the coupling coefficient becomes larger, thereby enalrging the FBW. Moreover, it can be captured from Figure 8b that, as *a*1 becomes wider, the in-band isolation becomes better, whereas as *a*2 becomes wider, the in-band isolation becomes worse.

**Figure 9:**
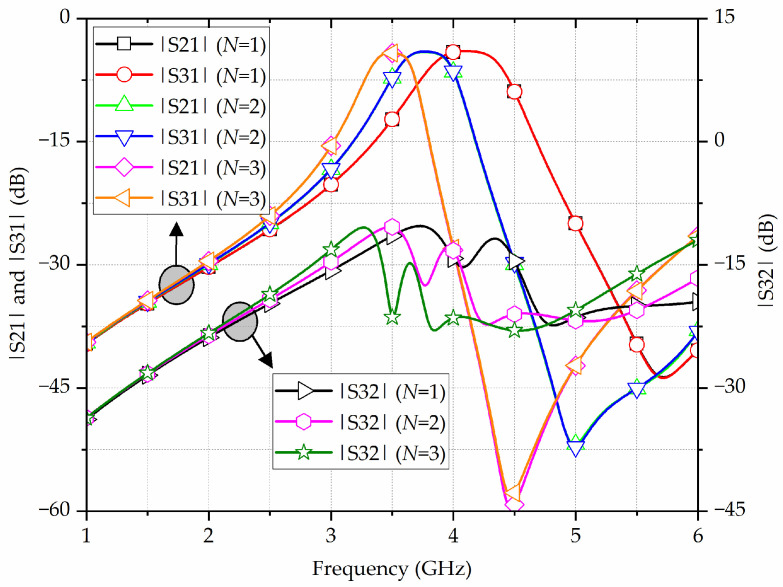
Simulated results of the proposed broadside-coupled SIW-IMSCSRR filtering power divider with various numbers of mender-shaped slots *N*.

Additionally, the influence of the number of mender-shaped slots *N* on the transmission properties of the proposed filtering power divider is given in Figure 9. It can be easily captured that, as *N* increases from 1 to 3, the center frequency moves downwards from 4.1 GHz to 3.5 GHz, while the bandwidth is squeezed from 450 MHz to 290 MHz, corresponding to the FBW decreasing from 10.97% to 8.28%. Obviously, with more mender-shaped slots, the center frequency can be lowered owing to the enhancement in both the equivalent capacitance and inductance. Moreover, with an increasing number of mender-shaped slots, both the coupling coefficient and the FBW decreases. Meanwhile, as *N* increases, the in-band isolation becomes better.

Finally, the proposed broadside-coupled SIW-IMSCSRR filtering power divider is simulated and optimized by using the same aforementioned full-wave electromagnetic simulator. The final optimized dimensions of the proposed broadside-coupled SIW-IMSCSRR filtering power divider are as follows: *w* = 10, *l* = 7, *wt*1 = 4, *lt*1 = 1, *wt*2 = 1.5, *lt*2 = 1.05, *x*1 = 0.6, *y*1 = 0.9, *x*2 = 0.6, *y*2 = 1, *a*1 = 2.2, *a*2 = 2.1, *lx*1 = 3.15, *ly*1 = 3.15, *lx*2 = 3.2, *ly*2 = 3.2, *wc*1 = 0.5, *lc*1 = 6, and *wm* = 0.94 (unit: mm).

## 4. Experiments and Discussion

**Figure 10:**
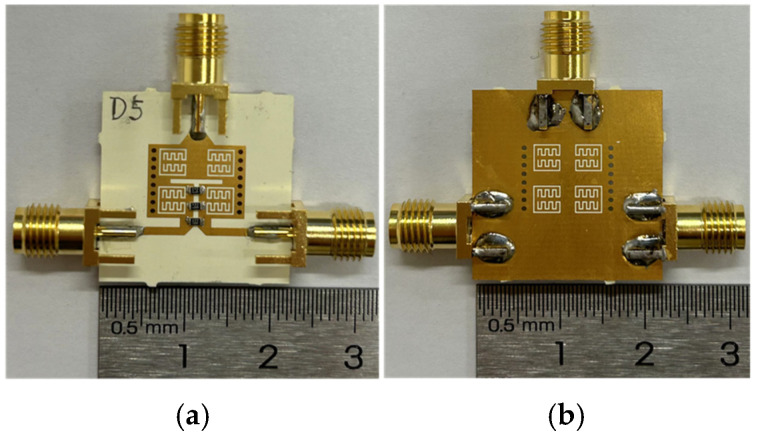
Photograph of the fabricated broadside-coupled SIW-IMSCSRR filtering power divider: (**a**) top view; (**b**) bottom view.

Using a standard printed circuit board process, a prototype of the proposed broadside-coupled SIW-IMSCSRR filtering power divider is fabricated. The substrate for fabrication is Rogers Kappa 438, with a thickness of 0.508 mm, a relative permittivity of 4.38, and a dielectric loss tangent of 0.005. The substrate is covered with lossy copper with a thickness of 0.035 mm. Moreover, the fabricated filtering power divider is plated with a gold surface of 2 um thickness to prevent oxidation of the copper cover. Finally, subminiature version A (SMA) connectors with a 50 ohm characteristic impedance are immediately soldered to the input and output 50 ohm microstrip lines for measurement convenience. A photograph of the fabricated broadside-coupled SIW-IMSCSRR filtering power divider is shown in Figure 10.

**Figure 11:**
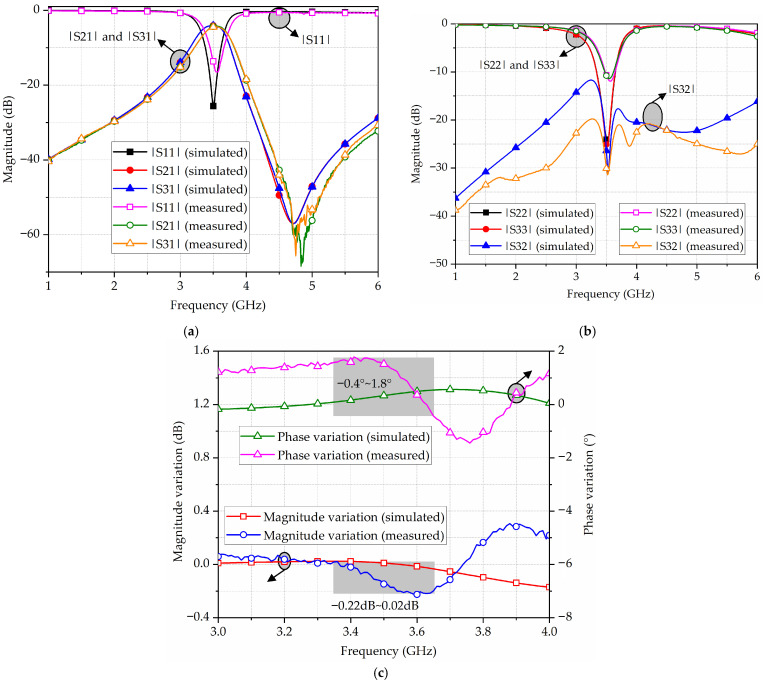
Simulated and measured results of the fabricated broadside-coupled SIW-IMSCSRR filtering power divider: (**a**) |S11|, |S21|, and |S31|; (**b**) |S22|, |S33|, and |S32|; (**c**) magnitude and phase variations.

The fabricated broadside-coupled SIW-IMSCSRR filtering power divider is measured using a Ceyear 3671D vector network analyzer (Qingdao, China), with simulated and measured results shown in Figure 11. At the beginning of measurement, the short–open–load–thru calibration method is utilized to remove the influence of the coaxial cables. Hence, the measurement results contain losses from the microstrip-to-SIW transitions and the 50 ohm microstrip lines at the input and output ports. As shown in Figure 11, the measured center frequency is 3.53 GHz, which is about 30 MHz higher than the simulated one. The measured insertion loss is (3 + 1.3) dB and the measured return loss is better than 16 dB. Meanwhile, a transmission zero appears around 4.8 GHz, with a suppression value of about 65 dB. Additionally, the in-band isolation is better than 21 dB, the magnitude variation ranges from −0.22 dB to 0.02 dB, and the phase variation ranges from −0.4°~1.8°. Moreover, the physical size of the fabricated broadside-coupled filtering power divider, including transitions, is about 11 mm × 10 mm, corresponding to a relative electrical size of 0.067 *λ*_0_^2^/*ε_r_*, where *λ*_0_ is the wavelength in free space and *ε_r_* is the relative permittivity of the substrate. In fact, compared to the top-loaded and bottom-loaded cases, the proposed broadside-coupled SIW-IMSCSRR filtering power divider can achieve a size reduction of about 30%.

To better demonstrate the overall performance of the fabricated broadside-coupled SIW-IMSCSRR filtering power divider, Table 1 summarizes comparisons with previously reported SIW filtering power dividers. Compared with the circular SIW cavity filtering power divider in [9], the right triangle SIW cavity filtering power divider in [10], the reconfigurable SIW cavity filtering power divider in [14], the tunable SIW cavity filtering power divider in [16], and the multilayered SIW filtering power divider in [22], the proposed one shows smaller insertion loss, smaller phase variation, much better isolation, and similar magnitude variation. Although the quarter-mode folded SIW (QMFSIW) filtering power divider with a stepped-impedance stub-loaded resonator (SISLR) in [23] exhibits better isolation than the proposed one, it shows larger insertion loss. Meanwhile, the magnitude and phase variations in [23] are not given. Furthermore, compared with the proposed work, the former three filtering power dividers in [24] achieve lower insertion losses but exhibit larger magnitude and phase variations, whereas the latter three filtering power divider designs in [24] exhibit lower insertion loss with similar magnitude and phase variations. However, all six filtering power dividers in [24] show limited isolation owing to the lack of specific isolation design considerations. Therefore, their isolation performance is limited. Hence, it can be obtained that the proposed broadside-coupled SIW-IMSCSRR filtering power divider achieves a good balance between insertion loss, magnitude and phase variations, and isolation. Finally, as shown in Table 1, compared with all the reported SIW filtering power dividers in [9,10,14,16,22,23,24], the proposed one achieves the most compact size. Specifically, compared to the six works in [24] and the one in [23], the proposed broadside-coupled SIW-IMSCSRR filtering power divider achieves size reduction of 75.9%, 73.7%, 65.2%, 64.3%, 50.3%, 66.9%, and 74.2%, respectively. Hence, based on the comparison in Table 1, the proposed filtering power divider achieves the smallest size while maintaining a good balance of overall performance. 

## 5. Conclusions

This work presents a broadside-coupled SIW-IMSCSRR filtering power divider. With the IMSCSRR and the broadside-coupling scheme, the equivalent capacitance and inductance of the SIW-IMSCSRR unit cell can be effectively enhanced, thus decreasing the fundamental resonant frequency significantly, and eventually achieving size reduction. Experimental results show that the proposed filtering power divider achieves good performance on insertion loss, isolation, and magnitude and phase variations. More importantly, compared with some similar reported works, the proposed broadside-coupled SIW-IMSCSRR filtering power divider achieves size reduction of over 50%. The proposed broadside-coupled SIW-IMSCSRR filtering power divider shows quite good potential for future microwave system integration applications.

## Figures and Tables

**Table 1:** Comparisons between some reported SIW filtering power dividers and the proposed one.

Ref.	Topology	Poles	*f*_c_ (GHz)	FBW (%)	Insertion Loss (dB)	Magnitude Variation (dB)	Phase Variation (°)	Isolation (dB)	Size (*λ*_0_^2^/*ε_r_*)
[9]	Circular SIW cavity	2	9.97	1	3 + 3.2	0.2	2	/	1.345
[10]	Right triangle SIW cavity	2	14.91	2.7	3 + 1.3	0.2	2.0	16.1	0.419
[14]	SIW cavity with microfluidic channel	1	3.67~5.2	11.5~17.2	3 + 1.61	/	/	15.34	0.216
[16]	Square/triangular SIW cavities	3	2.38~2.42	1.6~1.7	3 + (1.8~2.9)	0.5~0.57	5.2~8.6	>20	3.653
[22]	Multilayered SIW cavity	3	10.2	4	3 + 2	0.7	4	>5	1.02
[24]-1	Single-layered EMSIW + QMSIW	2	4.8	20.8	3 + 0.4	0.5	3.7	/	0.279
[24]-2	Single-layered EMSIW + QMSIW	2	4.86	20.6	3 + 0.6	0.5	3.6	/	0.255
[24]-3	Single-layered EMSIW + QMSIW	2	4.82	16.6	3 + 0.4	0.3	4.8	/	0.193
[24]-4	Single-layered EMSIW + QMSIW	2	4.8	16.6	3 + 0.6	0.15	1.6	/	0.188
[24]-5	Double-layered EMSIW + QMSIW	2	4.84	14.5	3 + 0.5	0.3	1.3	/	0.135
[24]-6	Double-layered EMSIW + QMSIW	2	4.83	12.4	3 + 0.6	0.2	1.4	/	0.203
[23]	QMFSIW + SISLR	4	4.98	15.2	3 + 1.47	/	/	25.2	0.260
**This work**	**Broadside-coupled SIW-IMSCSRR**	**2**	**3.53**	**9.1**	**3 + 1.3**	**−0.22~0.02**	**−0.4~1.8**	**>21**	**0.067**

## Data Availability

The original contributions presented in the study are included in the article, further inquiries can be directed to the corresponding author.

## Abbreviations

The following abbreviations are used in this manuscript:

SIW	Substrate-Integrated Waveguide
QMSIW	Quarter-Mode Substrate-Integrated Waveguide
EMSIW	Eighth-Mode Substrate-Integrated Waveguide
HMSIW	Half-Mode Substrate-Integrated Waveguide
CSRR	Complementary Split-Ring Resonator
IMSCSRR	Inner-Meander-Slot Complementary Split-Ring Resonator
PSR	Percentage of Size Reduction
FBW	Fractional Bandwidth
QMFSIW	Quarter-Mode Folded Substrate-Integrated Waveguide
SISLR	Stepped-Impedance Stub-Loaded Resonator
